# A Genome-Wide siRNA Screen Implicates Spire1/2 in SipA-Driven *Salmonella* Typhimurium Host Cell Invasion

**DOI:** 10.1371/journal.pone.0161965

**Published:** 2016-09-14

**Authors:** Daniel Andritschke, Sabrina Dilling, Mario Emmenlauer, Tobias Welz, Fabian Schmich, Benjamin Misselwitz, Pauli Rämö, Klemens Rottner, Eugen Kerkhoff, Teiji Wada, Josef M. Penninger, Niko Beerenwinkel, Peter Horvath, Christoph Dehio, Wolf-Dietrich Hardt

**Affiliations:** 1 Institute of Microbiology, Eidgenössische Technische Hochschule Zurich, CH-8093, Zurich, Switzerland; 2 Biozentrum, University of Basel, CH-4056, Basel, Switzerland; 3 Department of Neurology, University of Regensburg, DE- 93040, Regensburg, Germany; 4 Department of Biosystems Science and Engineering, Eidgenössische Technische Hochschule Zurich, CH-4058, Basel, Switzerland; 5 SIB Swiss Institute for Bioinformatics, 4058, Basel, Switzerland; 6 Division of Gastroenterology and Hepatology, University Hospital Zurich, University of Zurich, CH-8091, Zurich, Switzerland; 7 Zoological Institute, Technische Universität Braunschweig, D-38106, Braunschweig, Germany; 8 Department of Cell Biology, Helmholtz Centre for Infection Research, D-38124, Braunschweig, Germany; 9 Institute of Molecular Biotechnology of the Austrian Academy of Sciences (IMBA), A-1030, Vienna, Austria; 10 Light Microscopy Center, Eidgenössische Technische Hochschule Zurich, CH-8093, Zurich, Switzerland; University of Louisville, UNITED STATES

## Abstract

*Salmonella* Typhimurium (*S*. Tm) is a leading cause of diarrhea. The disease is triggered by pathogen invasion into the gut epithelium. Invasion is attributed to the SPI-1 type 3 secretion system (T1). T1 injects effector proteins into epithelial cells and thereby elicits rearrangements of the host cellular actin cytoskeleton and pathogen invasion. The T1 effector proteins SopE, SopB, SopE2 and SipA are contributing to this. However, the host cell factors contributing to invasion are still not completely understood. To address this question comprehensively, we used Hela tissue culture cells, a genome-wide siRNA library, a modified gentamicin protection assay and *S*. Tm^SipA^, a *sopBsopE2sopE* mutant which strongly relies on the T1 effector protein SipA to invade host cells. We found that *S*. Tm^SipA^ invasion does not elicit membrane ruffles, nor promote the entry of non-invasive bacteria "in trans". However, SipA-mediated infection involved the SPIRE family of actin nucleators, besides well-established host cell factors (WRC, ARP2/3, RhoGTPases, COPI). Stage-specific follow-up assays and knockout fibroblasts indicated that SPIRE1 and SPIRE2 are involved in different steps of the *S*. Tm infection process. Whereas SPIRE1 interferes with bacterial binding, SPIRE2 influences intracellular replication of *S*. Tm. Hence, these two proteins might fulfill non-redundant functions in the pathogen-host interaction. The lack of co-localization hints to a short, direct interaction between *S*. Tm and SPIRE proteins or to an indirect effect.

## Introduction

*Salmonella enterica* subspecies enterica serovar Typhimurium (*S*. Tm) is an enteroinvasive bacterial pathogen that represents one of the leading causes for human gastroenteritis. Infections occur via the oral route through ingestion of contaminated food or water. Common symptoms include diarrhea, nausea, vomiting and abdominal pain. The invasion of gut epithelial cells plays a central role in the infection cycle of *S*. Tm [1, 2]. Infections in healthy people are mostly self-limiting, but can be life-threatening for infants, elderly and immunocompromised patients. Because of the readily available tools to genetically manipulate *S*. Tm, it has become a model organism for invasive bacteria [3]. In order to improve therapies against *Salmonella* spp. and other pathogenic bacterial pathogens, it is of high importance to gain detailed understanding of their infection processes.

Several enteropathogenic bacteria can invade the gut epithelium [3]. Traditionally, the invasion strategies have been classified by morphological characteristics. *Yersinia* and *Listeria* spp. employ the so-called zipper mechanism, which hinges on adhesin-receptor interactions eliciting limited localized actin polymerization at the edges of membrane protrusions tightly wrapping around the bacteria. *Shigella* and *Salmonella* spp. invade via the trigger mechanism. Here, the injection of type 3 secretion system effector proteins triggers prominent actin rearrangements. Massive membrane ruffles mediate the macropinocytotic uptake of the pathogen and can even facilitate the internalization of otherwise non-invasive bacteria [3]. However, the host cellular factors contributing to the invasion are still not completely understood.

*S*. Tm is an enteropathogenic model-pathogen invading via the trigger mechanism. The SPI-1 type 3 secretion system (T1) allows docking to the host cellular surface [4, 5] and facilitates the injection of 15 T1 effector proteins [6–8]. SopE, SopE2, SopB and SipA are of central importance for triggering *S*. Tm host cell invasion. SipA can directly bind to actin and stabilizes actin filaments [9–15]. SopB is an inositol phosphatase while SopE and SopE2 are molecular mimics of host cellular G-nucleotide exchange factors activating host cellular RhoGTPases, i.e. RAC1 and CDC42 [16, 17] [11, 14, 18, 19]. *S*. Tm mutants lacking *sipA*, *sopB*, *sopE* and *sopE2* do not invade HeLa-like host cells but are incapable of eliciting overt mucosal inflammation *in vivo* [20]. In contrast, *S*. Tm mutants harboring only one of these four T1 effector proteins do retain appreciable virulence [11, 14, 20, 21]. Subsequent work has established substantial crosstalk or cooperativity between these different effectors. The concerted action of SopE and SopE2 leads to the subsequent recruitment of the WAVE regulatory complex (WRC) which is further activated, through a mechanism involving a set of ArfGTPases by SopB [22–24]. The WRC in turn drives actin rearrangement via the Arp2/3 complex [25–27]. Recent work has identified additional pathways of actin polymerization, e.g. via the Arp2/3-complex activator WASH [28] or the formin family actin nucleator FHOD1 [29]. Moreover, invasion can also occur through a myosinII-dependent contraction mechanism, operating downstream of Rho [30]. Taken together, the current model of *Salmonella*-driven actin reorganization during entry events involves different direct and indirect mechanisms that may act together to ensure efficient entry but may be redundant in their effects, at least partially. This was confirmed in two recent large-scale RNAi screens focusing on the role of SopE in *S*. Tm host cell invasion, which also identified central roles for COPI in RhoGTPase localization and of autophagy in T1-inflicted damage to the *Salmonella* containing vacuole (SCV;[31, 32]). In combination with approaches assessing the pathogen-mediated modulation and architecture of the host cellular actin cytoskeleton [8, 33–36], this has allowed substantial progress. Nonetheless, the interplay between the pathogen's and the host cellular factors is still not completely understood.

Recently, the SPIRE family has emerged as a class of host cell factors that may affect the invasion process. Mammalian SPIRE1 and SPIRE2 proteins cooperate with formin proteins (FMN1, FMN2, INF2) in nucleating actin filaments at vesicle, endosomal and mitochondrial membranes [37–41]. The SPIRE proteins are targeted towards vesicles and endosomes by a FYVE-type zinc finger domain, which interacts nonspecifically with negatively charged membranes [42]. The specificity for SPIRE protein targeting is thought to be mediated by additional protein/protein interactions. SPIRE function has been implicated in a variety of different cellular processes, e.g. Rab11 exocytic vesicle transport [43]; spindle positioning for asymmetric cell division in mouse oocytes [38] and mitochondria division [41]. In mouse metaphase oocytes SPIRE1 and SPIRE2 were found to cooperate with formin-2 and myosin Vb in microtubule-independent long-range transport of Rab11 vesicles along F-actin tracks [39]. In addition a SPIRE function has been described in the biogenesis of endosomal carrier vesicles/multivesicular bodies [44] and in complex with Rab3A in invadosome formation [45]. In spite of their different expression patterns [46], the mammalian SPIRE1 and SPIRE2 proteins seem to serve equivalent molecular functions. Interestingly, Spire2 has recently been implicated in *Listeria monocytogenes* invasion [47]. However, it had remained unclear whether it might also affect invasion by *S*. Tm.

Here, we have used a comprehensive image-based high-content siRNA screen to identify host factors contributing to *S*. Tm host cell invasion. Such siRNA screens have recently been applied to a number of pathogens and could provide important novel insights into the basic host cellular processes during infection [32, 48–54]. Our analysis of *S*. Tm^SipA^ (SL1344, *sopBsopEsopE2*) identified roles for Spire1 and Spire2 in different steps of the infection process.

## Materials and Methods

### Scanning electron microscopy

HeLa cells were cultured on glass coverlips coated with 16nm of carbon. At the end of the infection, cells were fixed (4% glutaraldehyde, 0.1x PBS) for 5min at 37°C and at 4°C for 2h. After washing, postfixation (1% OsO_4_ in water) was performed for 1h. Subsequently, the cells were washed, incubated in 0.5% carbohydrazide, washed again and incubated for a second time in 1% OsO_4_ (30 min). Thereafter, dehydration was performed in a series of ethanol baths and critical point drying was done with acetone (CPD 030, Bal-Tec AG, Liechtenstein). The dried coverlsips were mounted on SEM aluminum sample holders and sputter-coated with 5nm platinum. Images were recorded at 2 kV with a Zeiss Gemini 1530 FESEM (Zeiss, Oberkochen, Germany).

### Cell lines

HeLa CCL-2 cells (ATCC) (used for all screens except genome-wide unpooled Qiagen for SipA-invasion) and Kyoto (used for genome-wide unpooled Qiagen screen for SipA-invasion) were grown in DMEM (Gibco) supplemented with 10% inactivated FCS (Invitrogen) and 50 μg/ml streptomycin (AppliChem) at 37°C and 5% CO_2_.

### Cell and bacterial culture

HeLa and immortalized mouse embryonic fibroblast cell lines were grown in DMEM (Gibco) supplemented with 10% FCS (Omnilab) and 50mg/l streptomycin (AppliChem) at 37°C and 5% CO_2_. Mouse embryonic fibroblasts (*spire1*^gt/gt^ iMEF, *spire2*^-/-^ iMEF and suitable control lines) were derived from genetically modified *spire1* mutant mice [55] and yet unpublished spire2 knock out mice, which were generated by targeted deletion of exon 3, 4 and 5. Primary mouse embryonic fibroblast cells were immortalized sing SV40 large T-antigen [56, 57].

### siRNA libraries and transfection (genome wide screen)

The genome scale library was purchased from Qiagen and consisted of different subsets: HsDg 3.0 (27,000 siRNAs), HsNm1.0 and HsXm 1.0 (65,000 siRNAs) including at least 4 oligos per gene. For the Qiagen genome-wide screen, siRNA transfection was performed by seeding HeLa Kyoto cells into wells containing transfection reagents. 384-well plates (Matrix) had been preloaded with siRNA in 15μl water to yield a final concentration of 20nM and stored at -20°C. The transfection reagent was applied prior to cell seeding. Lipofectamine2000 (Invitrogen Inc.) was diluted 1:200 in Opti-MEM (Gibco) and after 15 minutes incubation at room temperature, 10μl were added to each well. Afterwards, 35μl DMEM (supplemented with 10% FCS) containing 700 cells were pipetted into each well and the plates were incubated for 3 days in a tissue culture incubator (37°C, 5% CO_2_ and saturated humidity).

Efficient transfection was monitored using the following controls: Hs_KIF11_7, Hs_PLK1_2 (transfection controls), Hs_ACTR3_8, Hs_ARPC3_5, Hs_CDC42_7, Hs_ATP1A1_7, Hs_CFL1_1 and Hs_ITGAV_7 (knock down controls; Qiagen).

### siRNA transfection

For siRNAs a reverse transfection protocol was used. In 96-well plates (μ-clear bottom, Greiner Bio One), 2 μl of 1 mM siRNA was added to 8 ml Opti-MEM (Invitrogen) yielding a final siRNA concentration of 20nM (after addition of cells). Lipofectamine 2000 (Invitrogen) was diluted 1:200 in Opti-MEM and incubated for 15 min at room temperature. A quantity of 10 μl per well were added and incubated for another 15 min at room temperature. These plates, hence forth referred to as cell plates, were either directly used or frozen at -80°C. HeLa cells were seeded using 1800–2000 cells in 80 μ well, followed by an incubation of 3 days at 37°C and 5% CO2. For half-size plates (μ-clear bottom, half area, Greiner Bio One), all numbers were reduced to 60%.

### Infection and automated modified gentamicin protection assay (genome wide screen)

HeLa Kyoto cells were infected with *S*. Tm^SipA^ harboring plasmid pM975 in a tissue culture incubator for 60min. Extracellular bacteria were killed 60minutes after the start of infection by medium replacement with DMEM containing 400μg/ml gentamicin (AppliChem). Bacteria expressed GFP only after successful internalization. Four hours post infection, cells were fixed with 4% paraformaldehyde in PBS (PFA, Sigma-Aldrich) and nuclei were stained using DAPI (1 or 10μg/ml, Sigma-Aldrich) in PBS containing 0.1% Triton X-100 (Sigma-Aldrich). For InfectX rescreening plates (all experiments besides the genome-wide screen), HeLa ATCC CCL-2 were used instead of HeLa Kyoto cell line. After fixation, cells were stained as described above but adding phalloidin-TRITC to stain for the actin cytoskeleton.

### Bacterial strains and plasmids

All *S*. Tm strains used were isogenic derivatives of SL1344 (SB300), a well-characterized strain of *Salmonella enterica* ssp. enterica serovar Typhimurium (*S*. Tm) [58]. All used *S*. Tm strains are shown in Table 1. Plasmid pM975 was used for T2-driven *gfp* expression in *S*. Tm. When *S*. Tm were used for infection into tissue-cultured cell-lines, they were grown in LB broth supplemented with 0.3M NaCl, 50 μg/ml streptomycin (Applichem) and ½ of the standard antibiotic concentration dependent on the harboring plasmids (like 50 μg/ml of Ampicillin in case of pM975;) for 12h at 37°C. These were diluted 1:20 in the same medium and sub-cultured for 4h at 37°C.

**Table 1:** Bacterial strains.

Designation	Strain	Genotype	Reference
*S*.Tm	SB300	SL1344 (wt)	[58]
*S*.Tm^SopE^	M701	Δ*sopE2*, *ΔsopB*, *ΔsipA*	[59]
*S*.Tm^SipA^	M516	Δ*sopE*, *ΔsopE2*, *ΔsopB*	[11]
*S*.Tm^Δeffectors^	M566	Δ*sopE*, *ΔsopE2*, *ΔsopB*, *ΔsipA*	[60]
*S*.Tm^ΔTTSS-1^	SB161	Δ*invG*	[61]
*S*.Tm^ΔTTTSS-1/Fim^	M556	Δ*invG*, Δf*imD*	[62]
*S*.Tm^ΔTTTSS-1/Fim^	SB161 pHR355	Δ*invG + Inv*	[62]
*S*.Tm^SipA-TEM^	M1128	*sipA-M45-TEM1*, Δ*sseD*	[31]
*S*.Tm^SipA-TEM/ΔTTSS-1^	M1114	*sipA-M45-TEM1*, Δ*invC*	[63]
p*rpsM*-*gfp*	pM965	*rpsM-gfp*	[64]
pT2-*gfp*	pM975	*pssaG-egfp*	[52, 65]

### Infection step assays

Binding, effector injection, modified gentamicin protection assay (GFP spot assay) and replication assay were performed as previously described [52, 66].

### *S*. Tm infection of siRNA screening plates (standard InfectX protocol)

For infection of siRNA screening plates, we used *S*. Tm^SipA^ (M516) harboring pM975 (Hapfelmeier et al., 2005). To perform the infection, a 4h subculture was used (see bacterial strains and plasmids) that reached late exponential growth phase (OD600nm≈1.0). Here 16μl of *S*. Tm^SipA^ (moi = 80, diluted in DMEM) were added to the HeLa cells and incubated for 60min at 37°C and 5% CO2. Afterwards, the *S*. Tm^SipA^ containing medium was replaced with 60μl DMEM/10% FCS containing 50μg/μl streptomycin and 400μg/μl gentamicin to kill all remaining extracellular bacteria. The cells were further incubated for 3h at 37°C and 5% CO2 and then fixed by adding 35μl 4% PFA, 4% sucrose in PBS for 20min at RT. The fixation solution was replaced by 60μl PBS containing 400 μg/ml gentamicin. Permeabilization of cells was performed for 5min with 40μl 0.1% Triton-X-100, which was replaced by 24μl of staining solution containing 1 μg/ml DAPI (Sigma-Aldrich) and 1.2 U/ml DY-547-phalloidin (Dyomics) in blocking buffer (4% BSA and 4% sucrose in PBS). After an incubation time of 1h at RT, the plates were washed three times with PBS and finally stored with 60 μl PBS containing 400 μg/ml gentamicin (sealed with Platesealer; Greiner bio one). All liquid handing stages of infection, fixation, and immunofluorescence staining were performed on a liquid handling robot (BioTek; EL406).

### Microscopy of screening plates

The 384-well screening plates were imaged in an automated fashion using the Molecular Devices ImageXpress microscope. Robotic plate handling was used to load and unload the plates. The objective used for acquisition was a 10X S Fluor with 0.45NA. 9 sites per well were imaged in a 3x3 grid without spacing or overlap of the images with three channels for monitoring the cell's nuclei (DAPI stain), the cell's actin cytoskeleton (DY-547-phalloidin) and the GFP-expressing bacteria (pM975).

### Data analysis

All data that were generated during InfectX related screens including raw and processed image data were shared through the openBIS biology information system [67]. To achieve an efficient automated image-analysis pipeline applicable to the distinct screening features exploited by the pathogens of the InfectX consortium, an open-source workflow management called screeningBee workflow manager was developed (Emmenlauer et al., unpublished). This workflow manager can modularly apply many image analysis steps in a reproducible manner. To allow for quantitative assessment of a very broad set of cellular and subcellular features on several segmented cellular compartments, several novel or enhanced image analysis and data normalization modules have been implemented based on CellProfiler [68] into the modular and generic image analysis framework of screeningBee [69]. Here the analysis involved image correction through the normalization of pixel intensities and shading correction of images before object detection using CellProfiler was performed. First, the "Nuclei" were detected in the DAPI channel using the IdentifyPrimAutomatic module of CellProfiler. In a 2^nd^ step the "PeriNuclei" was defined by an 8 pixel comprising extension of the nucleus object and the CellProfiler modules ExpandOrShrink and IdentifyTertiary were used to remove the nuclear area from this extended region. The "Cell" was identified according to its actin cytoskeleton surrounding the nucleus object (actin channel) using the BeeIdentifySecondaryInformed module. In addition, there was also a non-actin based cell defined through the extension of 25 pixels from the nucleus termed "VoronoiCells" which was used for infection scoring in the SipA genome wide Qiagen screen. On all four segmented objects (Nuclei, Perinuclei, Cells, VonoroiCells) more than 500 distinct features involving spatial, intensity and texture measurements were extracted. To detect the cells that were infected by *S*. Tm, a wavelet-based object detection was used to segment the GFP-dots that compose candidate locations of bacteria within the host cell. On the segmented bacteria candidates, a novel CellProfiler module (BeeMeasureObjectSubCell) was used to measure spatial features and the GFP intensity, resulting in a quantificative assessment of *S*. Tm infection. To discriminate and remove false positives of segmented Bacteria objects, a classification method based on a decision tree classifier has been applied. The decision tree classification has been optimized by a human expert, and identifies true internalized bacteria, and labels the parent cells as infected. This is the standard method to detect infection, applied to our presented data. To achieve best possible quality control of automated image-analysis for the siRNA screens, a second method to score infection was applied. Here infected cells were identified via CellClassifier [70] using supervised machine-based learning and a Support Vector Machine (SVM) classifier. As readout, the infection index defined the number of infected cells divided by the total numbers of cells in a well. To correct for potential plate or batch effects within siRNA screens, a non-control based z-scored data normalization was performed to normalize for variations between distinct plates. After plate z-scoring, the whole screen was z-score normalized in order to generate comparable data.

### KEGG pathway mapping of the hits

Hits (gene median of -0.5 or lower) were mapped on all KEGG pathways using the open source R/bioconductor package pathview [71].

### RT-qPCR

Cells were washed with PBS, displaced from the flask using Trypsin EDTA and spun down at 1000xg for 5min. The supernatant was resuspended in 600 μl RLT buffer. Total RNA extraction was done using the RNeasy mini kit (Qiagen) with RNase-free DNase digest (Qiagen). For reverse-transcription of 1 μg mRNA aliquots, the RT^2^ HT First Strand cDNA Kit (Qiagen) was used. Custom RT^2^ Profiler PCR Arrays (Qiagen) were run with RT^2^ SYBR Green ROX FAST (QIAGEN) on an Applied Biosystems 7900 HT Fast Real-Time PCR System to amplify the resulting cDNA. Relative mRNA levels (2^−ΔCq^) were determined by comparing the PCR quantification cycle (Cq, determined with the Software SDS 2.2.1) with the reference gene *ACTB*. The differences in their Cq cycles were calculated (ΔCq). In all experiments, the upper limit of Cq was fixed to 35 cycles.

### Immunofluorescence staining, image acquisition and quantification

HeLa cells were seeded on coverslips 1 day before transfection and transfected with pcDNA3.1 expression vectors containing HA-tagged genes of interest using Lipofectamine2000 (Invitrogen). One day after transfection, cells were infected with *Salmonella* strains at MOI = 50, as indicated. At time points of interest, cells were washed twice with PBS and fixed using 2% PFA (Sigma) buffered in PBS for 15min at room temperature. Cells were permeabilized in 0.1% Triton-X-100 (0.1% Triton-X-100, 0.05% NaAz in PBS) and blocked in 3% BSA (3% BSA with 4% sucrose in PBS). For staining, we used a mouse anti-HA antibody diluted (1:500, Roche) in 3% BSA and incubated for 1h at RT in a wet chamber. The primary antibodies were washed away thrice with PBS and the secondary staining was carried out with an anti-mouse Cy5 antibody (1:600, Jackson Immuno) in 3% BSA, DAPI (1:1000) and phalloidin-TRITC (1:600) for 1h at room temperature. Cells were subsequently washed three times with PBS and the coverslips were mounted on microscope slides with MOWIOL mounting media. Images were acquired on a Zeiss Axiovert 200m inverted microscope with a Visitron confocal system and a PLAN-Apochromat 100x oil objective (Zeiss, NA 1.3). To analyze co-localization, images were quantitatively analyzed using the plot profile function on a line perpendicularly drawn through a bacterium in Fiji (ImageJ) and normalized to the highest fluorescence within the measurement.

### Statistics

Statistical differences between cell lines were analyzed using a Mann-Whitney test. Significance has been indicated as follows: * P < 0.05, ** P < 0.01, *** P < 0.001 and **** P <0.0001.

### Gene enrichment analysis

Gene Set Enrichment Analysis (GSEA) was performed to test for the enrichment of known biological pathways based on two ranked gene lists: top 300 (short) and 2670 hits (long). We used the GSEAPreranked module from the Broad Institute GSEA suite with default parameters, a minimum set size of 10 and 1000 permutations. The enrichment was tested against the Canonical Pathway database (c2.cp.v4.0.entrez.gmt) from MSigDB. Enriched pathways are visualised separately for each list using a heat map representation where the color indicates the false discovery rate (FDR).

## Results

### Genome-wide RNAi screen using *S*. Tm^SipA^

**Fig 1:**
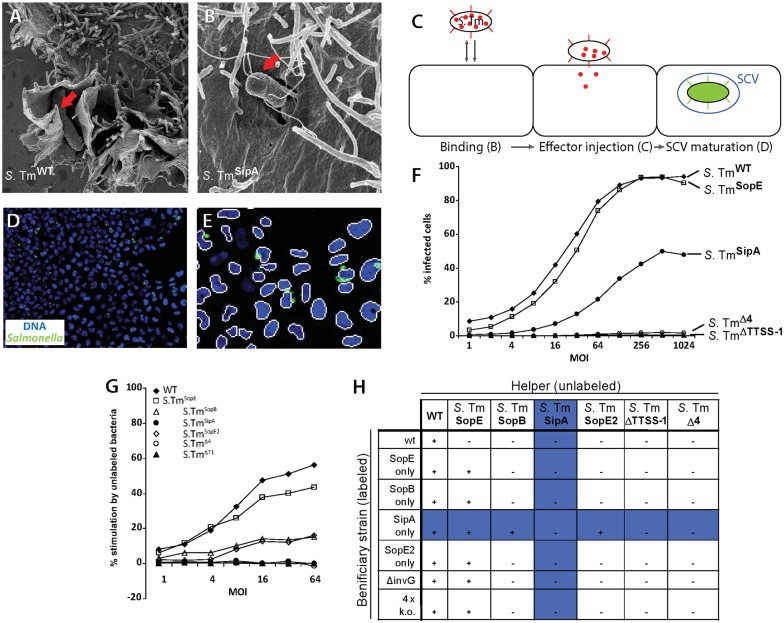
(A and B) Scanning electron micrographs of wild type (A) and *S*. Tm^SipA^ strains (B) infecting HeLa cells. (C) Representation of *Salmonella* Typhimurium infection steps (adapted from **[31]**). (D) Representative image of the genome-wide RNAi screen, (E) spot- and nuclei-detection of the infectX pipeline (right) at MOI150. (F) HeLa Kyoto cells were infected at increasing MOI using different SPI-1 effector mutant strains (Table 1) and the infection efficiency infection was monitored using the modified gentamicin protection assay. (G) Helper assay using different T1 effector mutants in HeLa cells. (H) Representation of helper activity of different T1 effector mutants.

To screen for host cell factors contributing to host cell invasion, we have used *S*. Tm^SipA^ (M516; SL1344 *sopBsopEsopE2*; [11]; Table 1). This strain was chosen, as it lacks the key T1 effectors known to trigger the archetypical membrane ruffles [11, 16, 17, 72–75]. Therefore, it should allow for sensitive detection of host cell factors, the contribution of which is masked by the ruffling response triggered by SopE, SopB and SopE2. Indeed, upon infection of HeLa cells, *S*. Tm^SipA^ did not elicit pronounced membrane ruffling (Fig 1A and 1B), but was still invading with appreciable efficiency, as indicated by a modified gentamicin protection assay. The latter assay employs a GFP reporter under control of the *ssaG*-promotor (Fig 1C–1F; T2-*gfp* reporter; [31, 32]; Materials and Methods). Thus, *gfp* is expressed by *S*. Tm only after successful invasion and the maturation of the SCV. This allowed us to quantify the fraction of infected cells by automated microscopy and -image analysis. The ruffle-less nature of *S*. Tm^SipA^ invasion was verified in a helper assay which quantitatively assesses how a given *S*. Tm strain of interest affects host cell invasion by other *S*. Tm strains. In this type of assay, HeLa cells are infected with a mixture of the helper strain (w/o GFP reporter) and a second *S*. Tm strain of interest which carries the T2-*gfp* reporter (Fig 1G). In line with earlier work, wt *S*. Tm promoted the invasion by all other *S*. Tm strains tested, including *S*. Tm^SipA^, ΔinvG (SB161; non-invasive due to a disrupted T1 apparatus) and *S*. Tm^Δ4^ (M516; SL1344 *sopBsopEsopE2sipA*; [60]), an isogenic strain which is non-invasive by itself (Fig 1H). This helper effect is attributable to the pronounced membrane ruffling triggered by wt *S*. Tm [32, 52]. In contrast, *S*. Tm^SipA^ did not affect the invasion efficiency of any of the strains, analyzed (Fig 1H). This provided additional evidence that *S*. Tm^SipA^ invasion occurs in the absence of pronounced membrane ruffling and indicated that the associated host cellular membrane-rearrangements do only support bacterial invasion in cis.

**Fig 2:**
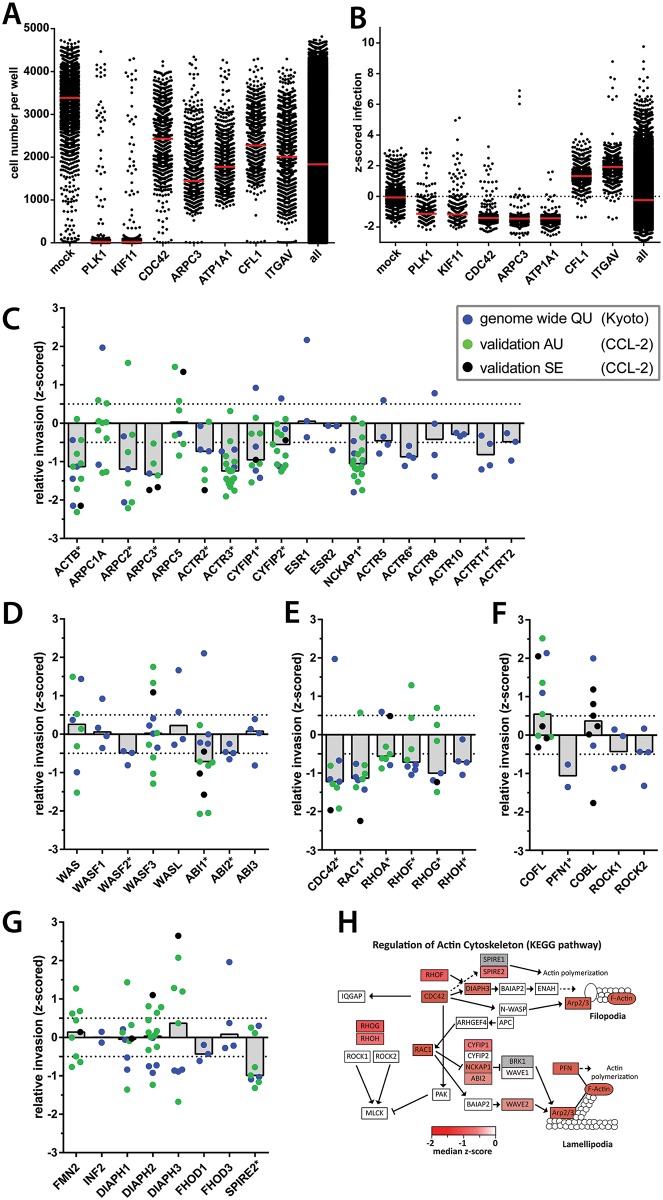
(A) Quality control 1 of the genome wide screen. Cell numbers of chosen controls throughout the whole genome wide screen. (B) Quality control 2 of the genome wide screen. Infection index of chosen controls throughout the whole genome wide screen. Red bars = median of all data points. (C-G) Results of the genome-wide screen are functionally grouped. Color of data points indicates the type of experiment and type of RNAi used. QU = Qiagen unpooled siRNA in HeLa Kyoto cells (blue; original screen). Additional data for knockdown of the presented genes are indicated in green or black: AU = Ambion unpooled siRNA in HeLa CCL-2 cells (green). SE = Sigma esiRNA in HeLa CCL-2 cells (black). Bars represent median of all data points. Asterisks after gene names indicate hit for genome wide QU screen. Panels show proteins indicated in ARP2/3 complex and regulation (C), WAVE, WASH, WASP and interacting proteins (D), RhoGTPases (E), additional actin interacting proteins and regulators (F) and formins (G). (H) KEGG pathway mapping of decreasing hits from the Qiagen unpooled *S*. Tm^SipA^ siRNA screen. Hits reducing the fraction of infected cells below -0.5 median z-score infection index were mapped on all KEGG pathways. KEGG pathway for regulation of actin cytoskeleton is shown. Color code: Strength of the observed phenotype (median z-score). White fields: no hits or no data available from the genome-wide screen. Grey fields were not included in the library or had to be excluded due to low cell number. Adapted from Kreibich et al., 2015.

Using the gentamicin protection assay, we performed genome-wide RNAi screening. HeLa Kyoto cells were transfected with the Qiagen siRNA library (unpooled, 3–4 oligos per gene; https://pubchem.ncbi.nlm.nih.gov/bioassay/1117357), infected with *S*. Tm^SipA^ (T2-*gfp*), fixed and stained with DAPI, as described above. The fraction of infected cells was analyzed in an automated fashion (>500cells analyzed per siRNA; Materials and methods). Z-scored normalization of individual screening plates ensured best possible comparison between plates and screens. The data are available online [https://pubchem.ncbi.nlm.nih.gov/bioassay/1117357]. The quality of the screen was controlled by including siRNAs with "known" effects on each plate of the screen. The efficiency of siRNA transfection was controlled using siRNAs knocking down the crucial cell cycle regulators PLK1 and KIF11. This is expected to yield mitotic arrest, apoptosis and cell death [76]. Indeed, the cell number was significantly reduced for the large majority of the control wells (median number of nuclei; PLK1 = 23; KIF11 = 55). In contrast to PLK1 and KIF11, the other controls and the vast majority of library-siRNAs did not affect the numbers of cells by more than 2-fold (Fig 2A; https://pubchem.ncbi.nlm.nih.gov/bioassay/1117357). This was of importance, as 2-fold changes in cell number did not affect the infection efficiency per se (pilot data; not shown). Plates retaining >500 cells per PLK1 or KIF11 control well were excluded from further analysis. To validate the quality of the infection screening, we used siRNAs targeting genes known to affect *S*. Tm invasion. Knocking down CDC42 (-1.58 median z-score invasion) or ARPC3 (-1.58 median z-score invasion), the Na^+^/K^+^ exchanger (ATP1A1: -1.43 median z-score; [31]) reduced while knocking down CFL1 (1.40 median z-score invasion) or ITGAV (1.59 median z-score invasion) promoted infection, as shown in earlier work on *S*. Tm^SopE^ [31]. The latter effect is likely attributable to a rounded cell morphology, significantly increasing bacterial binding and thereby promoting infection, as described recently [5].

The screen of the genome-wide Qiagen library and several targeted siRNA libraries identified 2671 hits with a mean z-scored infection index of ≤0.5, which is estimated to correspond to >30% infection efficiency (https://pubchem.ncbi.nlm.nih.gov/bioassay/1117357; S1 Fig). Re-discovery of signaling modules of known importance for *S*. Tm host cell invasion further supported the quality of the screen. This included components of the COPI complex (COPG,COPB2; https://pubchem.ncbi.nlm.nih.gov/bioassay/1117357; [31]), components of the autophagy-machinery which promotes the repair of T1-damaged SCV membranes (https://pubchem.ncbi.nlm.nih.gov/bioassay/1117357; [32]), endosome acidification (https://pubchem.ncbi.nlm.nih.gov/bioassay/1117357; [31, 32]) and numerous cellular factors controlling the regulation and the architecture of the host cellular actin cytoskeleton (Fig 2C–2H; https://pubchem.ncbi.nlm.nih.gov/bioassay/1117357). The analysis of functional and interactive protein clusters using STRING- [77] and DAVID [78] and mapping of the siRNA phenotypes to the KEGG-pathways of interest further illustrated the central importance of the actin cytoskeleton in *S*. Tm^SipA^ invasion (Fig 2H). Interestingly, this group of hits also included SPIRE2, an actin nucleator that has not yet been widely characterized [79]. Mammalian cells encode two homologues of the SPIRE family of actin nucleators, namely SPIRE1 and SPIRE2, which are expressed in different tissues. However SPIRE1, the second member of the family, is not covered by the Qiagen library. Thus, our original screen only provided data for SPIRE2. SPIRE2 has recently been found to affect *L*. *monocytogenes* invasion via the zipper mechanism [47]. However, this actin nucleator has not yet been implicated in *S*. Tm infection. Our screening data provided a first indication that this class of actin nucleator might contribute to one or more steps of the *S*. Tm infection process.

### Verification of the SPIRE phenotypes

**Fig 3:**
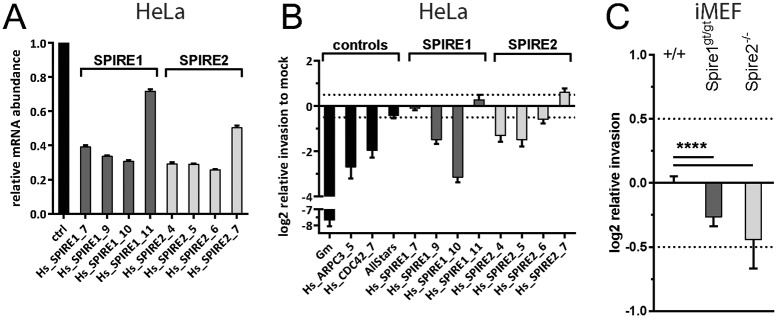
(A) *SPIRE1* and *SPIRE2* expression as measured by RT-qPCR upon RNAi treatment. Columns represent mean and error bars show standard deviation. (B) Small scale validation screen using *S*. Tm^SipA^ and the modified gentamicin protection assay to confirm phenotype of SPIRE2. (C) Modified gentamicin protection assay using *Spire1*^gt/gt^ and *Spire2*^-/-^ iMEFs infected with *S*. Tm^SipA^ normalized to the wild type iMEF control cell line. A shows data from 2 independent experiments with 2 replicates each in HeLa Kyoto cells. B and C show data from 3 independent experiments with 3 replicates each. Asteriscs indicate significant differences. *: p<0.05.

In order to substantiate the importance of the SPIRE family proteins, we followed two strategies. In a first set of experiments, we employed four different siRNAs per gene in order to knock down *SPIRE1* (Qiagen siRNA SI03225355, SI04260529, SI04279044, SI04657933) or *SPIRE2* (Qiagen siRNA SI00732032, SI04225179, SI04227349, SI04351305) expression in HeLa Kyoto cells and quantified the effects on gene expression (Fig 3A). As commercial antibodies did not yield reliable signals in Western blot analyses, we used quantitative rtPCR assays to verify knockdown. This revealed that SPIRE1 oligos 7, 9 and 10 and the SPIRE2 oligos 4, 5 and 6 significantly reduced the expression of *SPIRE1* or *SPIRE2*. In parallel, we performed infection assays with *S*. Tm^SipA^ (pT2-*gfp*), as described above. Strikingly, all siRNA which reduced SPIRE mRNA levels also reduced the efficiency of *S*. Tm^SipA^ invasion (Fig 3B). The knockdown with the oligo SPIRE1_7 was the only exception. Such "deviating" phenotypes are frequently observed in siRNA screens and are often attributable to "off-target" effects which can mask the expected phenotype [80, 81]. Overall, however, these observations supported roles for SPIRE2 (and possibly SPIRE1) in *S*. Tm^SipA^ host cell invasion.

In a second approach, we made use of immortalized mouse embryonic fibroblast (iMEF) cell lines that were derived from mice carrying a terminator (gene trap, gt) between exons 3 and 4 of the *spire1* [55] or the *spire2* knock out mouse, generated by a deletion of exons 3, 4 and 5 of the mouse *spire2* gene (provided by J. Penninger). These cells therefore lack functional Spire1 or -2 proteins. Primary fibroblasts were isolated from homozygous, gene-targeted or wildtype, control mice, and immortalized to obtain iMEFs (see methods). Both knockout cell lines showed significantly lower levels of invasion by *S*. Tm^SipA^ (pT2-*gfp*) than the wt iMEF control (p<0.05; Fig 3B). However, it should be noted that we have not analyzed if knockdown or ablation of SPIRE1 or -2 affects (directly or indirectly) the expression of other cellular components known to control *Salmonella* host cell invasion (e.g. Cdc42, Arp2/3 etc). Neverthless,our data verified that Spire2 and (to a lesser extent) Spire1 proteins are of importance for *S*. Tm^SipA^ invasion

### SPIRE1 and SPIRE2 affect different stages of the *S*. Tm infection process

The modified gentamicin protection assay using the T2-*gfp* reporter will detect defects in SCV maturation or all stages of the infection process which are preceding this step [31, 32]. Thus, it had remained unclear which steps were affected by the SPIRE proteins and if SPIRE2 and SPIRE1 affect the same step of *Salmonella* host cell invasion. To address this question, we performed a series of infection-step-specific assays using the wt and knockout iMEFs.

**Fig 4:**
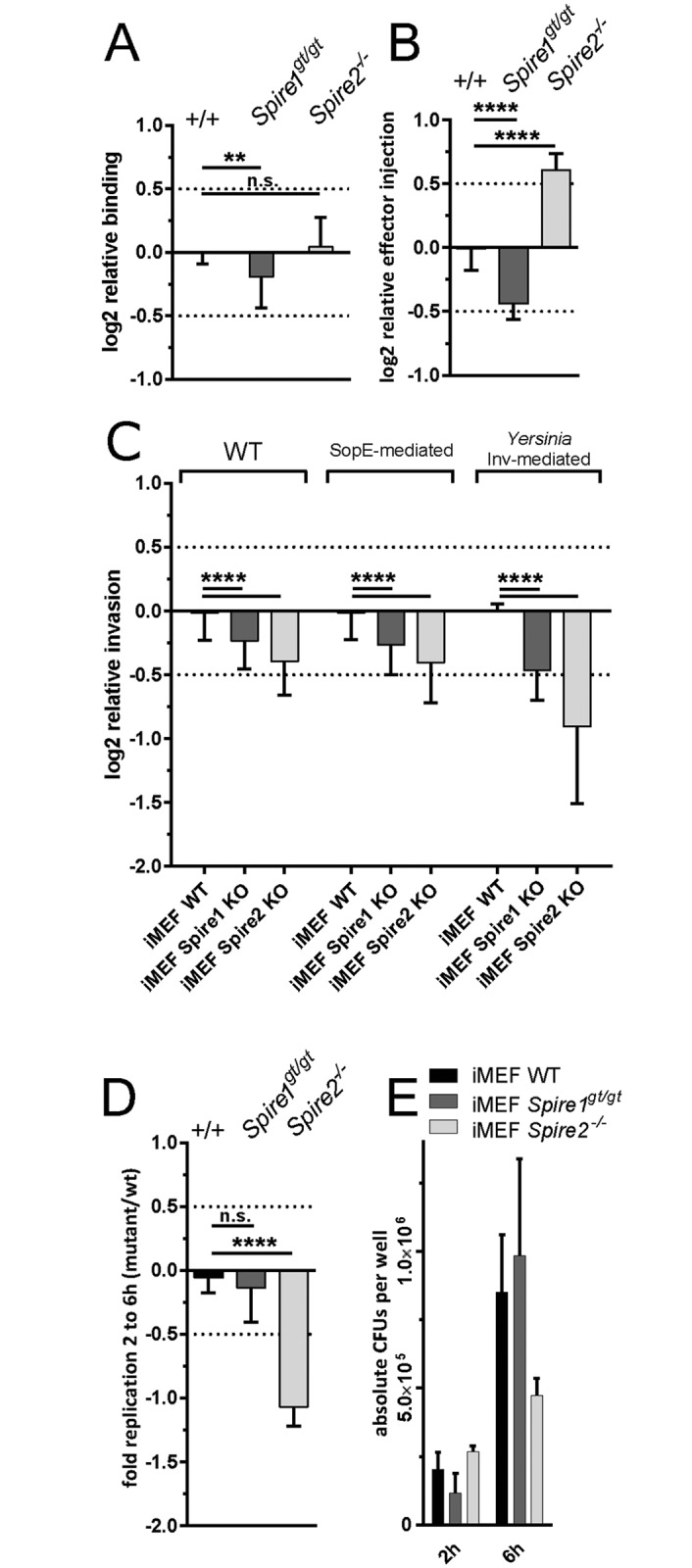
Infection step assays using iMEF cell lines. (A) Binding assay using *Spire1*^gt/gt^ and *Spire2*^-/-^ iMEFs normalized to the data from the wild type iMEF control cell line. *Spire1*^gt/gt^ show reduced *Salmonella* binding. (B) Effector injection assay using *Spire1*^gt/gt^ and *Spire2*^-/-^ iMEFs normalized to the wild type iMEF control cell line. *Spire1*^gt/gt^ and *Spire2*^-/-^ cells show attenuation and increase in effector injection, respectively. (C) Modified gentamicin protection assay using *Spire1*^gt/gt^ and *Spire2*^-/-^ iMEFs normalized to the wild type iMEF control cell line. *Spire1*^gt/gt^ and *Spire2*^-/-^ cell lines show decrease in invasion after 4h. Invasion is not dependent on *Salmonella* effectors or invasion type. (D) Intracellular replication in *Spire1*^gt/gt^ and *Spire2*^-/-^ iMEFs normalized to the wild type iMEF control cell line measure by plating assay. *Spire2*^-/-^ cell line shows decrease of intracellular bacterial replication. (E) Absolute number of CFUs corresponding to D. A-C show data from 3 independent experiments with 2 replicates each in HeLa Kyoto cells. D and E show data from 2 independent experiments with 3 replicates each. Asteriscs indicate significant differences. *: p<0.05.

To assess binding, we infected wt iMEF controls, *Spire1*^gt/gt^ or *Spire2*^-/-^ cells with *S*. Tm^Δ4^, a mutant featuring a functional T1 apparatus, but lacking the four invasion-mediating SPI1 effector proteins SipA, SopE, SopE2 and SopB. Therefore, this strain can still dock to the host cell surface, but fails to trigger invasion [4, 32, 52]. *Spire2*^-/-^ iMEF showed similar levels of *S*. Tm^Δ4^ binding as wt iMEF (p>0.05; n = 3 Independent assays; Fig 4A), while binding to *spire1*^gt/gt^ iMEF was slightly reduced (p<0.05).

To assess T1-mediated effector protein translocation, we used *S*. Tm^Δ4sipA-TEM^ (M1128; SL1344 *sipA*::*sipA-M45-TEM1 sseD*::*aphT sopAsopBsopEsopE2*). Upon binding and T1-mediated injection of SipA-TEM into the host cellular cytoplasm, the TEM-fusion protein will cleave a β-lactamase sensitive substrate (CCF-2AM) that had been loaded into the cells. This cleavage-associated shift in the fluorescence spectrum of the infected cell can be detected by fluorescence spectroscopy and serves as a sensitive readout for the efficiency of T1 effector protein translocation [52, 63, 82]. Strain M1114 (SL1344, *sipA*::*sipA-M45-TEM1*, *invC*::*aphT*) which features a defective T1 apparatus served as a fluorescence background control. *S*. Tm^Δ4sipA-TEM^ showed reduced levels of SipA-TEM translocation in the *Spire1*^gt/gt^ iMEF (p<0.05; Fig 4B) and increased levels of SipA-TEM translocation in the *Spire2*^-/-^ cells as compared to the wt iMEF (p<0.05; Fig 4B).

Host cell invasion was analyzed using the pT2-*gfp* reporter assay. To address how SPIRE-deficiency affects not only *S*. Tm^SipA^ (which invades w/o triggering pronounced membrane ruffles), but also wt *S*. Tm and *S*. Tm^SopE^ (SL1344, *ΔsipA ΔsopB ΔsopE2*), two strains invading by triggering pronounced membrane ruffles. *S*. Tm^ΔT1Invasin^ (SL1344 Δ*invG*, carries Inv-expression plasmid), an isogenic strain with a defective T1 apparatus which can invade via the Invasion protein from *Yersinia enterocolitica* (facilitates invasion via the zipper mechanism; [20]), served as an additional control. All four *S*. Tm strains feature reduced invasion efficiencies in the *spire1*^gt/gt^ iMEF (p<0.05; Fig 4C). The attenuation of invasion was even more pronounced in the the *Spire2*^-/-^ iMEF (p<0.05; Fig 4C). This indicated that SPIRE-family proteins have a general effect on *S*. Tm invasion, no matter whether ruffling is involved or whether the bacteria invade via a classical "zipper" adhesin.

Finally, we analyzed the rate of intracellular replication. This was done by infecting fibroblasts for 60 minutes at a low MOI, washing the cells and adding medium containing gentamicin to kill bacteria that were not internalized (Materials and Methods). Cells were permeabilized after 1 or 5 additional hours of infection and cell contents plated on bacterial growth agar. The resulting colony forming units were used to bacterial replication rates between 1h and 5h after internalization (corresponding to 2h and 6h p.i.). Intracellular replication of *S*. Tm^SipA^ was significantly reduced in *Spire2*^-/-^ iMEF, but not in *Spire1*^gt/gt^ cells (p<0.05; Fig 4D). Together, these data established that SPIRE1 has a significant (though weak) effect on pathogen binding (Fig 4A) which may also explain the reduced effector injection (Fig 4B) efficiency and the reduced overall rate of invasion into the siRNA-treated HeLa Kyoto cells or the *Spire1*^gt/gt^ iMEF (Figs 3C/4D). In contrast, SPIRE2 has little effect on pathogen binding, but (directly or indirectly) limits effector protein injection, SCV formation and intracellular replication. Thus, SPIRE1 and SPIRE2 seem to contribute to different steps of the *S*. Tm infection processes.

### Co-localization analysis of SPIRE proteins with *Salmonella* Typhimurium

**Fig 5:**
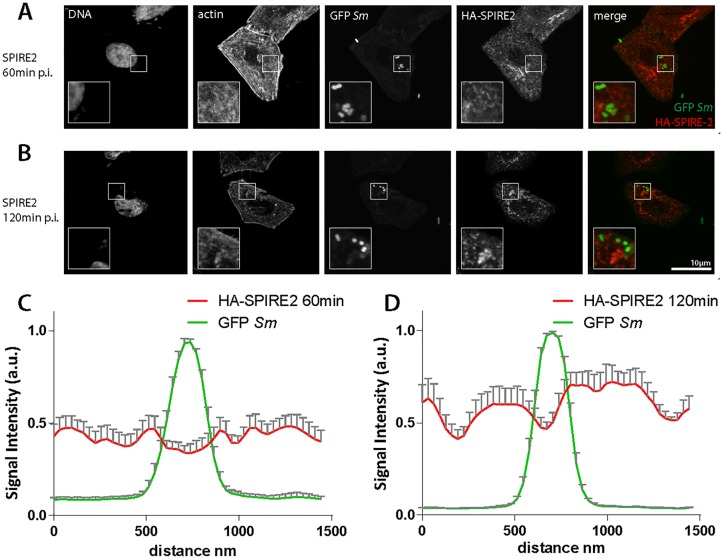
SPIRE1 and SPIRE2 do not co-localize with *Salmonella* Typhimurium after invasion. (A and B) SPIRE2 overexpressing HeLa cells infected with *Salmonella* Typhimurium. SPIRE2 does not co-localize with bacteria as shown in merge. (C and D) Quantification of immunofluorescence images as shown in A and B. Same phenotype was observed for SPIRE1 in two different cell lines and two different *Salmonella* Typhimurium strains. No co-localization observed after 15min (data not shown).

So far, it had remained unclear how SPIRE proteins affect *S*. Tm infection. It was conceivable that direct interactions with adhesins or SCV-associated effector proteins might be involved. We applied confocal immunofluorescence microscopy in order to find out whether SPIRE proteins localize to the bacteria during early infection events. HA-tagged versions of the human *SPIRE1* and *SPIRE2* cDNAs were cloned into mammalian expression vectors and transiently transfected into HeLa cells. These HeLa cells (or mock-transfected control cells) were subsequently infected for 60 or 120 minutes with *S*. Tm^SipA^ expressing GFP from a constitutive promoter. Infections with *S*. Tm^SopE^ served as an additional control. Cells were fixed and stained for DNA, actin and the HA-tag and imaged by fluorescence microscopy. The fluorescence signals in the green and red (HA-tag) channels in the vicinity of the bacteria was quantified using ImageJ [83]. Neither SPIRE1 nor SPIRE2 were enriched in the vicinity of the bacteria (e.g. the SCV membrane, a site where effector proteins like SopE can be localized for >60 min after invasion;[84]) neither at 60 nor at 120 min p.i. (Fig 5). These data exclude a prominent recruitment of the SPIRE proteins to the pathogen binding sites or the SCV. Further work will be required to decipher how exactly they contribute to the infection process.

## Discussion

Infection by many invasive bacteria is dependent on cytoskeletal rearrangements [85]. *Salmonella* Typhimurium and other invasive bacteria make use of a variety of endogenous host cell mechanisms to drive their internalization. Here we show that the *S*. Tm infection process is influenced by the recently discovered actin nucleating factors SPIRE1 and SPIRE2 [86] in cells of murine and human origin.

Knock down or deletion of *SPIRE1* led to a decrease of bacterial binding. In contrast, knockdown or deletion of *SPIRE2* affected SCV maturation or intracellular *S*. Tm replication. Interestingly, SPIRE2 (but not SPIRE1) has recently been shown to promote the efficiency of host cell infection by *Listeria monocytogenes* [47]. The molecular basis of this effect remains to be established. As SPIRE proteins have been implicated in vesicle transport processes [39, 43, 87], it is interesting to speculate that SPIRE2 may affect pathogen containing vacuoles or egress from the endosomal compartment. However, the intra-vacuolar steps of the *S*. Tm and the *L*. *monocytogenes* infection differ in some important aspects [3, 88]. In tissue culture models, *L*. *monocytogenes* is only transiently engulfed by a host cellular membrane and pathogen egress into the host cell cytosol occurs within ten minutes. In contrast, *S*. Tm remains within endosomal compartments for extended periods of time. Wild-type host cells limit *S*. Tm egressing into the cytoplasm in an autophagy-dependent fashion and the *Salmonella-*containing vacuole matures to induce expression of SCV-specific virulence factors, i.e. the SPI2 type III secretion system [32, 89]. Thus, if the role of SPIRE2 in *L*. *monocytogenes* and *S*. Tm infections roots in the same molecular process, one may speculate that the formation of the early endosome or the control of pathogen egress into the host cell cytoplasm might be modulated by SPIRE2.

SPIRE1 seems to have a different function than SPIRE2. This is indicated by knockdown experiments which failed to detect a role for SPIRE1, but not SPIRE2, in the *L*. *monocytogenes* infection of HeLa cells [47] and by the binding defect (which was not observed for SPIRE2) in the *S*. Tm infection (this work). It will be an interesting topic for future work to determine how SPIRE1 affects *S*. Tm binding. Earlier work had established that the insertion of the SPI1 translocon or the 3D topology of the host cellular surface can have profound effects on *S*. Tm binding [4, 5, 90, 91]. It remains to be shown if either of these host cellular functions are affected by SPIRE1. The requirement for SPIRE1 in *S*. Tm host cell invasion might be restricted to particular cell types. This is suggested by the tissue-specific gene expression pattern and by our observation that SPIRE1 knockdown affects *S*. Tm invasion into HeLa Kyoto cells, but not into the HeLa cell line CCL-2. At any rate, the functional differences detected between SPIRE1 and SPIRE2 in the HeLa Kyoto and the iMEF infection assays might be of general interest, as earlier studies of SPIRE proteins have not detected significant functional differences between the different family members [39, 87]. Additionally, it will be important to investigate a potential colocalization between *S*. Tm and SPIRE proteins that we were not able to demonstrate. Formally, we cannot rule out an indirect mechanism, i.e. SPIRE1/2 mediated modulation of other pathogen-host interactions.

*In vivo*, SPIRE1 and SPIRE2 show distinct expression patterns in the different tissues of young and adult mice [46]. *Spire1* is mainly expressed in neuronal tissues like cerebellum and cerebrum while *Spire2* is highly expressed in gastrointestinal tract of young and adult mice. Immunohistochemistry has detected particularly high SPIRE2 protein in the epithelium of the murine gut [92]. As these gut epithelial cells are the primary target for host cell invasion by *S*. Tm [1, 2], these observations suggest that SPIRE2 might also affect the infection *in vivo*. Infection experiments in *Spire* knock out mice may help to establish the role of SPIRE proteins in the infection process *in vivo*.

## Supporting Information

S1 Fig Gene Set Enrichment Analysis (GSEA) of the top 300 (short) and 2670 hits (long) of the genome-wide siRNA screen. The enrichment was tested against the Canonical Pathway database (c2.cp.v4.0.entrez.gmt) from MSigDB. Enriched pathways are visualised separately for each list using a heat map representation with the color indicating false discovery rate (FDR).
(TIF)
