# Supplementary figures and images for: A Genome-Wide siRNA Screen Implicates Spire1/2 in SipA-Driven Salmonella Typhimurium Host Cell Invasion
Source: PLoS One. 2016 Sep 14;11(9):e0161965. doi: 10.1371/journal.pone.0161965 (PMC5023170; doi:10.1371/journal.pone.0161965)

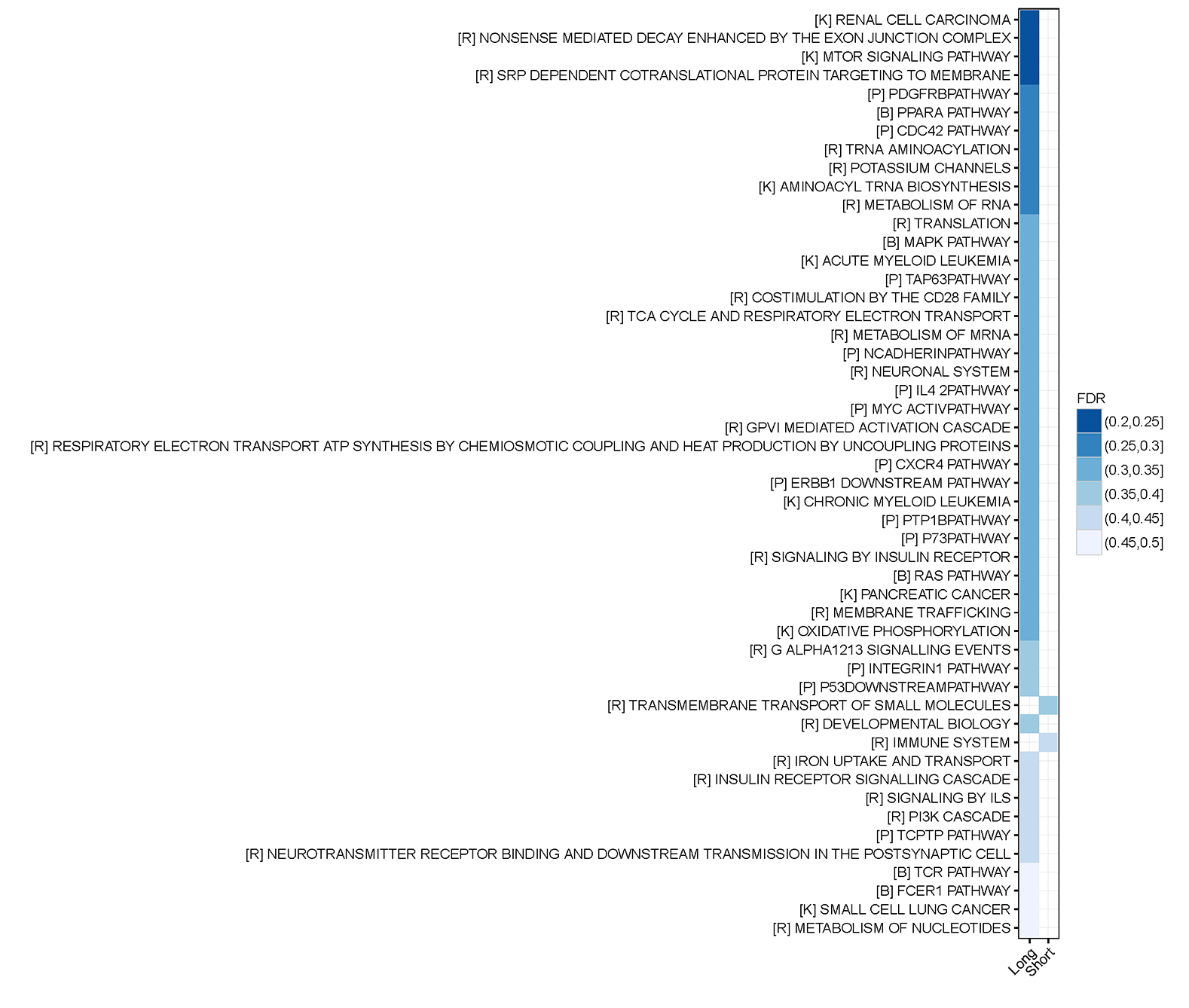

Supplement: S1 Fig — The enrichment was tested against the Canonical Pathway database (c2.cp.v4.0.entrez.gmt) from MSigDB. Enriched pathways are visualised separately for each list using a heat map representation with the color indicating false discovery rate (FDR). (TIF) [file pone.0161965.s001.tif]
